# Inhibition of the Transforming Growth Factor-β Signaling Pathway Confers Neuroprotective Effects on Beta-Amyloid-Induced Direct Neurotoxicity and Microglia-Mediated Neuroinflammation

**DOI:** 10.1155/nri/8948290

**Published:** 2025-01-30

**Authors:** Shao Qin Tiong, Raxshanaa N. Mohgan, Jia Yee Quek, Jennifer Yue Suan Liew, Grace Yee Seen Wong, Zi Qing Thang, Zhi Ling Chan, Sook Yee Gan, Elaine Wan Ling Chan

**Affiliations:** ^1^School of Pharmacy, IMU University, Bukit Jalil, Kuala Lumpur, Malaysia; ^2^Institute for Research, Development and Innovation, IMU University, Bukit Jalil, Kuala Lumpur, Malaysia

**Keywords:** Aβ-induced neurotoxicity and microglia-mediated neuroinflammation, Alzheimer's disease, TGF-β signaling

## Abstract

**Background:** Abnormal elevation of transforming growth factor-beta (TGF-β) has been observed among Alzheimer's disease (AD) patients. This may be due to microglia-mediated release of proinflammatory cytokines, which promote neuroinflammation and neuronal apoptosis. Silencing of *TGFBR1*, a gene encoding TGF-β receptor type I (TGF-βR1), has resulted in neuronal survival from amyloid-beta (Aβ)-induced neurotoxicity. Therefore, the present study investigated the neuroprotective effect of TGF-βR1 inhibitors (RepSox, Galunisertib, and Vactosertib) against Aβ-induced direct neurotoxicity and microglia-mediated neuroinflammation.

**Methods:** The neuroprotective effect of TGF-βR1 inhibitors against Aβ-induced direct neurotoxicity and microglia-mediated neuroinflammation were investigated using the RealTime-Glo™ MT Cell Viability Assay. The inhibitory effect of TGF-βR1 inhibitors on Aβ-induced microglia-mediated production of proinflammatory cytokines (TNF-α and IL-1β) was determined using enzyme-linked immunosorbent assay (ELISA).

**Results:** TGF-βR1 inhibitors (RepSox, Galunisertib, and Vactosertib) at the tested concentrations (6.25–150 nM) showed no significant cytotoxicity effects on SH-SY5Y and BV-2 cells. Moreover, treatments with these inhibitors exhibited neuroprotection on SH-SY5Y cells against Aβ-induced direct neurotoxicity. The trend of cell viability after 24 h treatment also supports the microscopic images of the cells' morphology. Furthermore, pretreatment with these inhibitors conferred indirect neuroprotective effect against Aβ-induced microglia-mediated neuroinflammation by attenuating the production of proinflammatory cytokines (TNF-α and IL-1β).

**Conclusion:** The inhibition of the TGF-β signaling pathway in neuronal and microglia cells by TGF-βR1 inhibitors resulted in neuroprotection against Aβ-induced direct neurotoxicity and microglia-mediated neuroinflammation. Hence, targeting the TGF-β signaling pathway in both neuronal and microglia cells could provide a promising therapeutic strategy in AD.

## 1. Introduction

Alzheimer's disease (AD), the most common type of dementia worldwide, is an irreversible, progressive neurodegenerative disorder and the most prevalent disease in the aging community [1]. Globally, around 47 million people have suffered from dementia. This figure is expected to double every two decades, reaching 131.5 million by 2050 [2]. Decades of research have established that the neuropathology of AD results from the accumulation of extracellular amyloid-beta (Aβ) peptides and intracellular neurofibrillary tangles (NFTs) of hyperphosphorylated tau (p-Tau) [3–5]. Recently, neuroinflammation mediated by microglial cells has shown to play a significant role in neurodegeneration [6–8]. Proinflammatory cytokines such as interleukin-1β (IL-1β), IL-6, tumor necrosis factor-alpha (TNF-α), as well as inflammatory mediators such as nitric oxide (NO) are released by microglial cells in response to triggers including Aβ plaque and aggregates of tau protein [8–11]. Under normal conditions, these cytokines exert neuroprotective effects by recruiting additional microglia to the inflammatory site to eliminate proinflammatory stimuli and dead cells via phagocytosis. However, the overproduction of these proinflammatory mediators by microglia leads to the neuroinflammation cascade, which eventually results in neuronal dysfunction and uncontrolled inflammation in neuronal cells. This process induces further microgliosis and neuronal death that drives the progression of AD [8, 12–17].

Although AD is considered as a global public health priority, only symptomatic treatments are currently available (donepezil, galantamine, rivastigmine, and memantine) with their demonstrated benefits on measures of behavior, daily function, and cognition [18]. These drugs mitigate the cognitive symptoms of AD without slowing disease progression. The lack of effective treatment regimens has prompted researchers to investigate new potential therapeutic targets for AD beyond the well-known neuropathological hallmarks [18, 19]. Using a gene trap mutagenesis approach, researchers have found that the *TGFBR1* gene is involved in Aβ-induced neurotoxicity, and silencing this gene had resulted in neuronal survival [20]. The *TGFBR1* gene encodes TGF-β receptor type 1 (TGF-βR1) that participates in signal transduction with TGF-β as the ligand [21]. Upon binding of TGF-β cytokines to TGF-β receptor type II (TGF-βR2), TGF-βR1, also known as activin receptor-like kinase 5 (ALK5), is phosphorylated by TGF-βR2. The resulting receptor complex phosphorylates the intracellular SMAD2 and SMAD3. Phosphorylated SMAD2 and SMAD3 accumulate into a heterotrimeric complex with SMAD4, acting as a transcription factor to activate various target genes in the nucleus [22–25]. In fact, TGF-β superfamily signaling plays a central role in inflammation and the brain's response to injury. Its dysregulation partakes significantly in the pathogenesis of neurological disorders, including AD [26, 27]. However, the role of TGF-β signaling in AD pathogenesis has been conflicting, whereby some studies have reported that TGF-β1 administration can restore synaptic plasticity and alleviate neurodegeneration [28, 29]. In addition, pretreatment of TGF-β1 could prevent the elevation of Aβ-induced proinflammatory mediators and regulate the expression of proapoptotic and antiapoptotic factors, leading to neuroprotection in AD models [28, 30]. On the contrary, abnormal elevation of TGF-β detected in AD brains has been reported to promote neuroinflammation and neuronal apoptosis [31, 32].

Interestingly, inhibition of TGF-β signaling, either via TGF-βR1 inhibitor (SB431542) or SMAD-3 knockdown, has shown to attenuate neurotoxicity induced by carbofuran, that impairs neurogenesis, by altering TGF-β signaling [33]. Numerous studies have highlighted the potential therapeutic applications of TGF-βR1 inhibitors for treating various disorders in which its progression has been associated with the TGF-β pathway [34–41]. Given the potential therapeutic properties of TGF-βR1 inhibitors in AD treatment, the present study aims to investigate the neuroprotective effect of selective TGF-βR1 inhibitors (RepSox, Galunisertib, and Vactosertib) against Aβ-induced neurotoxicity and Aβ-induced microglia-mediated neuroinflammation. In addition, the study will examine their inhibitory effect on the production of proinflammatory cytokines (IL-1β and TNF-α) in Aβ-induced microglia-mediated neuroinflammation.

## 2. Materials and Methods

### 2.1. Materials and Reagents

The SH-SY5Y neuroblastoma cell line (ATCC CRL-2266; passage 6) and BV-2 microglial cell line (passage 8) were purchased from the American Type Culture Collection (ATCC), USA. Dulbecco's modified Eagle medium (DMEM) was purchased from Corning Inc., USA. Galunisertib, RepSox, phosphate buffer saline (PBS), 0.25% trypsin-EDTA solution, phenylmethylsulfonyl fluoride (PMSF), protease inhibitor cocktail (PIC), and 2-mercaptoethanol were purchased from Sigma-Aldrich, USA. Vactosertib was obtained from 1PlusChem LLC, United States. Dimethyl sulfoxide (DMSO) and 3-(4,5-dimethylthiazol-2-yl)−2,5-diphenyltetrazolium bromide (MTT) reagent were obtained from Calbiochem, USA. Penicillin/streptomycin (P/S) and trypan blue stain (0.4%) were obtained from GIBCO/Life Technologies, USA. Fetal bovine serum (FBS) was purchased from Biosera, France. Synthetic amyloid-β (1–42) and RealTime-Glo™ MT Cell Viability Assay kit were purchased from Genscript, USA and Promega, USA, respectively. Mouse interleukin-1β and tumor necrosis factor-alpha enzyme-linked immunosorbent assay (ELISA) kits were purchased from Sigma-Aldrich, USA. Quick Start™ bovine serum albumin (BSA) standard, ammonium persulfate (APS), Quick Start™ Bradford 1 × dye reagent, Immun-Blot polyvinylidene fluoride (PVDF) membranes, 30% acrylamide/Bis solution 37.5:1 and 4 × Laemmli sample buffer were purchased from Bio-Rad, USA. Methanol and 10 × radioimmunoprecipitation assay (RIPA) buffer were, respectively, obtained from Friendemann Schmidt, Malaysia, and Abcam, UK. N,N,N′,N′-tetramethylethylenediamine (TEMED), Tween 20, sodium chloride, sodium dodecyl sulfate (SDS), tris base, and glycine were obtained from Fisher Scientific, USA. Hydrochloric acid and PM2510 ExcelBand™ Enhanced 3-color Regular Range Protein Marker were purchased from Merck KGaA, Germany and SmoBio Technology, Taiwan respectively. Blocking One and Chemi-Lumi One Super enhanced chemiluminescent (ECL) were obtained from Nacalai Tesque, Japan. Rabbit monoclonal anti-β-actin primary antibody, horseradish peroxidase (HRP)-linked secondary antibody anti-rabbit IgG, and HRP-linked secondary antibody anti-mouse IgG were purchased from Cell Signaling Technology, USA. Mouse monoclonal anti-Tau (Tau-5) primary antibody and anti-p-Tau (PHF-6) primary antibody were obtained from Santa Cruz Biotechnology, USA.

### 2.2. Preparation of Stock Solutions

A 5 mM stock solution of Aβ was prepared by dissolving the dried synthetic Aβ in DMSO. This stock solution was diluted in PBS to 200 μM and kept at room temperature for 3 h, followed by incubation at 4°C for 24 h. Stock solutions (1 mg/mL) of TGF-βR1 inhibitors (RepSox, Galunisertib, and Vactosertib) were prepared by dissolving the respective drugs in DMSO. All stock solutions were stored at −20°C until use.

### 2.3. Cell Culture

The human SH-SY5Y neuroblastoma cell line and the murine BV-2 microglia cell line were cultured in T25 and T75 flasks, respectively, with complete media (DMEM supplemented with 10% heat-inactivated FBS and 1% P/S) in a humidified cell incubator under a mixture of 95% air and 5% carbon dioxide at 37°C. For the direct neuroprotective assay, SH-SY5Y cells were seeded in six-well plates or 96-well plates at densities of 1 × 10^6^ cells/mL and 5 × 10^5^ cells/mL, respectively. For the indirect neuroprotective assay, BV-2 cells were seeded in six-well plates at a density of 4 × 10^5^ cells/mL, while SH-SY5Y cells were seeded in 96-well plates at a density of 5 × 10^5^ cells/mL. The seeded cells were allowed to stabilize for 24 h at 37°C before exposed to subsequent treatments in serum-free medium (SFM).

### 2.4. Cytotoxicity Effect of TGF-βR1 Inhibitors

SH-SY5Y cells and BV-2 cells were treated separately with TGF-βR1 inhibitors ranging from 6.25 nM to 150 nM for 24 h (the concentration range were selected with reference to the reported range of half-maximal inhibitory concentration (IC_50_)) of the TGF-βR1 inhibitors (RepSox, 23 nM [42]); Galunisertib: 56–172 nM [35, 43, 44] and Vactosertib: 11–16.5 nM [43, 45–47]. The vehicle control (VC) consisted of untreated cells cultured in media with 0.02% DMSO. After 24 h, the medium was removed from the plates, and the cells were incubated with 25 μL of 2 mg/mL MTT reagent for 4 h at 37°C. Then, the MTT medium was aspirated from the wells, and the purple formazan crystals formed were dissolved in 100 μL of DMSO. The absorbance was measured at 570 nm with a reference wavelength of 700 nm using a microplate reader (Tecan Spark™ 10 M, Switzerland). The results were expressed as the percent viability relative to the VC.

### 2.5. Neuroprotective Effect of TGF-βR1 Inhibitors Against Aβ-Induced Direct Neurotoxicity on SH-SY5Y Cells

SH-SY5Y cells were pretreated with TGF-βR1 inhibitors at 50 nM, 100 nM, and 150 nM, respectively (concentration selected based on cytotoxicity results and reported IC_50_), along with the RealTime-Glo™ reagents in SFM for 4 h. Next, the SH-SY5Y cells were exposed to 20 μM Aβ [48], and the luminescence signal was measured at specific time points over 48 h using a microplate reader (Tecan Infinite M200 Pro, Switzerland) set at 37°C. The results were expressed as the percent viability relative to the VC. The treatment/control groups are described in Table 1. The morphology of Aβ-induced SH-SY5Y cells pretreated with 100 nM TGF-βR1 inhibitors was observed at 100x magnification under phase contrast microscopy using an inverted brightfield microscope (Nikon Eclipse Ti-U, USA).

### 2.6. Neuroprotective Effect of TGF-βR1 Inhibitors on SH-SY5Y Cells Against Aβ-Induced Microglia-Mediated Neuroinflammation

A standardized concentration of 100 nM of the TGF-βR1 inhibitors was selected for the treatments involving microglial-mediated neuroinflammation. BV-2 cells were pretreated with 100 nM of TGF-βR1 inhibitors for 4 h, after which 2 μM of Aβ solution was added to the cultures for 24 h. Conditioned media (CM) from the BV-2 microglia cell cultures, along with RealTime-Glo™ reagents, were transferred to SH-SY5Y cells to replace the basal media (Table 2). The cell viability of SH-SY5Y cells was assessed using RealTime-Glo™ MT Cell Viability Assay.

### 2.7. Inhibitory Effects of TGF-βR1 Inhibitors on Aβ-Induced Proinflammatory Cytokines (TNF-α and IL-1β) Production in BV-2 Cells

The BV-2 cells were pretreated with 100 nM of TGF-βR1 inhibitors for 4 h, followed by exposure to 2 μM Aβ for 24 h [49]. Culture media containing 0.1% DMSO was used as the VC. The levels of proinflammatory cytokines (TNF-α and IL-1β) in the supernatant of cultured BV-2 cells were measured using ELISA kits according to the manufacturer's instructions. Data were normalized using Bradford assay and expressed in picograms of TNF-α per microgram of total protein and picograms of IL-1β per milligram of total protein. The treatment/control groups are shown in Table 3.

### 2.8. Statistical Analysis

All data were demonstrated as the mean ± standard error of the mean (SEM) of three independent experiments. Statistical analyses were performed using GraphPad Prism software version 8.0.2. Data were analyzed using one-way analysis of variance (ANOVA; normality test: Shapiro–Wilk test), followed by Tukey's post hoc test. Results were considered as statistically significant when the *p* value was less than 0.05.

## 3. Results

### 3.1. Neuroprotective Effect of TGF-βR1 Inhibitors Against Aβ-Induced Direct Neurotoxicity

**Figure 1:**
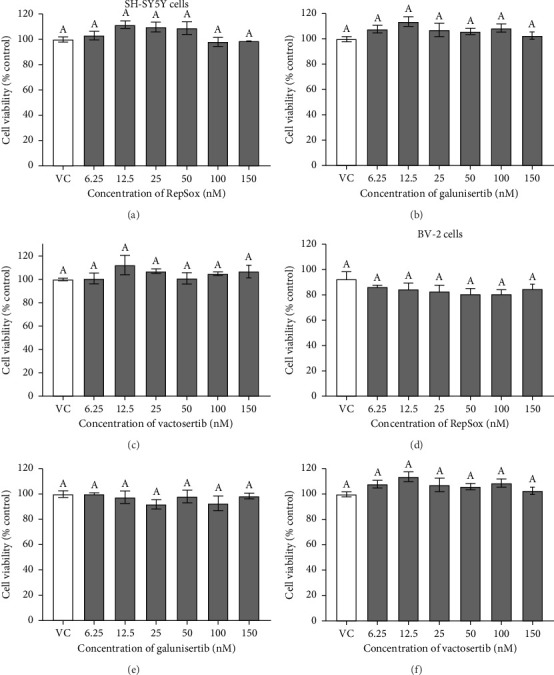
Cytotoxicity effect of TGF-βR1 inhibitors on SH-SY5Y neuroblastoma cells (a–c) and BV-2 microglial cells (d–f). The cells were exposed to a range of concentrations of RepSox (a, d), Galunisertib (b, e), and Vactosertib (c, f) from 6.25 nM to 150 nM. Vehicle control (VC) consisted of untreated cells culture in media containing 0.02% DMSO. The results were expressed as percentage cell viability versus VC. All values were shown as the mean ± SEM and representative of three independent experiments (*n* = 3). The treatment groups labeled with the same alphabets are not statistically significant (*p* > 0.05).

**Figure 2:**
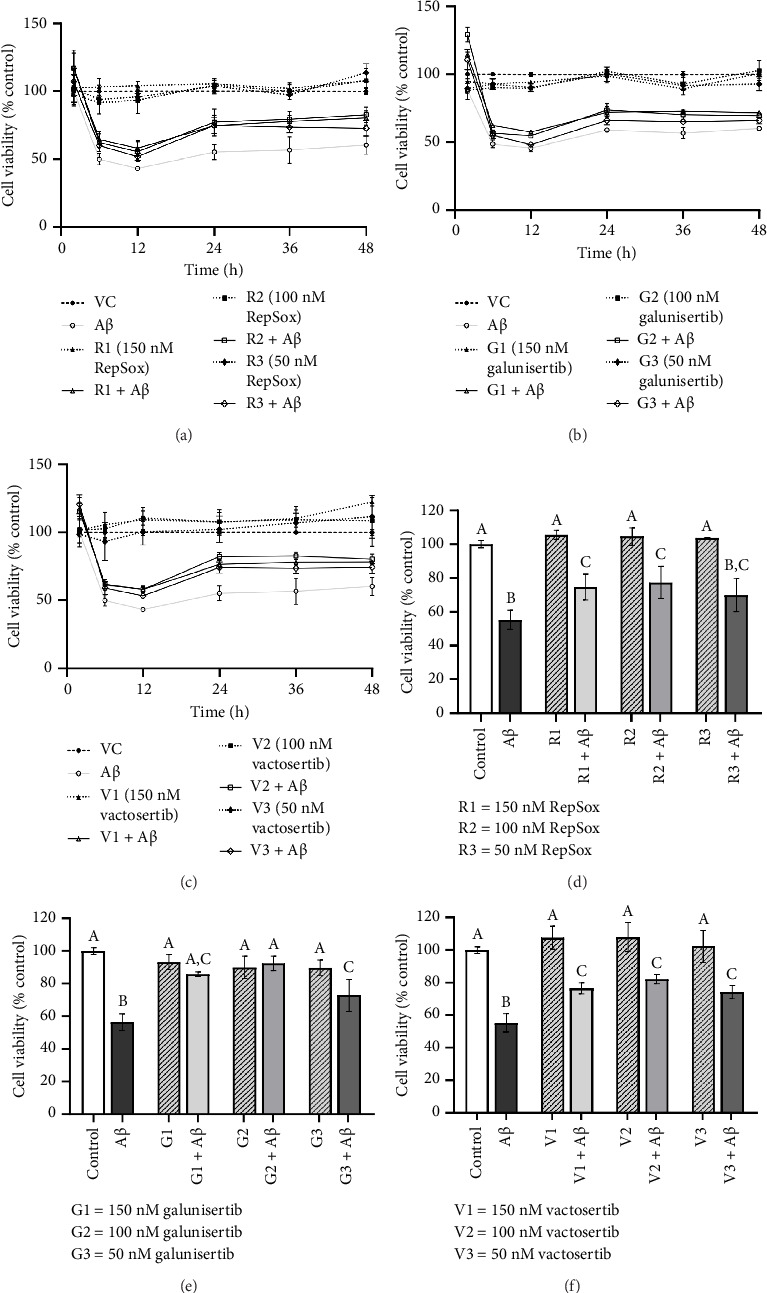
Neuroprotective effect of TGF-βR1 inhibitors on SH-SY5Y neuroblastoma cells against Aβ-induced neurotoxicity measured in real time over 48 h (a–c) and at the time point of *t* = 24 h (d–f). The cells were pretreated with three drug concentrations (1: 50 nM, 2: 100 nM and 3: 150 nM) of RepSox (R) ((a): *t* = over 48 h; (d): *t* = 24 h), Galunisertib (G) ((b): *t* = over 48 h; (e): *t* = 24 h) and Vactosertib (V) ((c): *t* = over 48 h; (f): *t* = 24 h), respectively, along with the addition of RealTime-Glo reagents for 4 h. The luminescent signal was measured after the addition of 20 μM Aβ at specific time points. Untreated cells cultured in media containing 0.02% DMSO were included as the vehicle control (VC). The results were expressed as percentage cell viability versus VC. All values were shown as the mean ± SD and representative of three independent experiments (*n* = 3). The treatment groups labeled with the same alphabets are not statistically significant (*p* > 0.05).

There were no significant cytotoxicity effects on SH-SY5Y and BV-2 cells when treated with the TGF-βR1 inhibitors at the tested concentrations (6.25–150 nM) compared with the VC (Figure 1). In the neuroprotective assay, SH-SY5Y cell viability was significantly decreased upon exposed to Aβ only, in comparison with VC (Figure 2). Interestingly, pretreatment with TGF-βR1 inhibitors (RepSox, Galunisertib, and Vactosertib) at all three tested concentrations (50 nM, 100 nM, and 150 nM) improved cell viability of Aβ-induced SH-SY5Y cells over the 48-h treatment (Figures 2(a), 2(b), and 2(c)). Importantly, after Aβ induction over 24 h, it was evident that pretreatment with TGF-βR1 inhibitors at all the tested concentrations, except for 50 nM RepSox, significantly improved the cell viability of SH-SY5Y cells, in comparison with the Aβ control group (Figures 2(d), 2(e), and 2(f); *p* < 0.05). These results indicate that all TGF-βR1 inhibitors conferred a neuroprotective effect against Aβ-induced neurotoxicity on SH-SY5Y cells.

**Figure 3:**
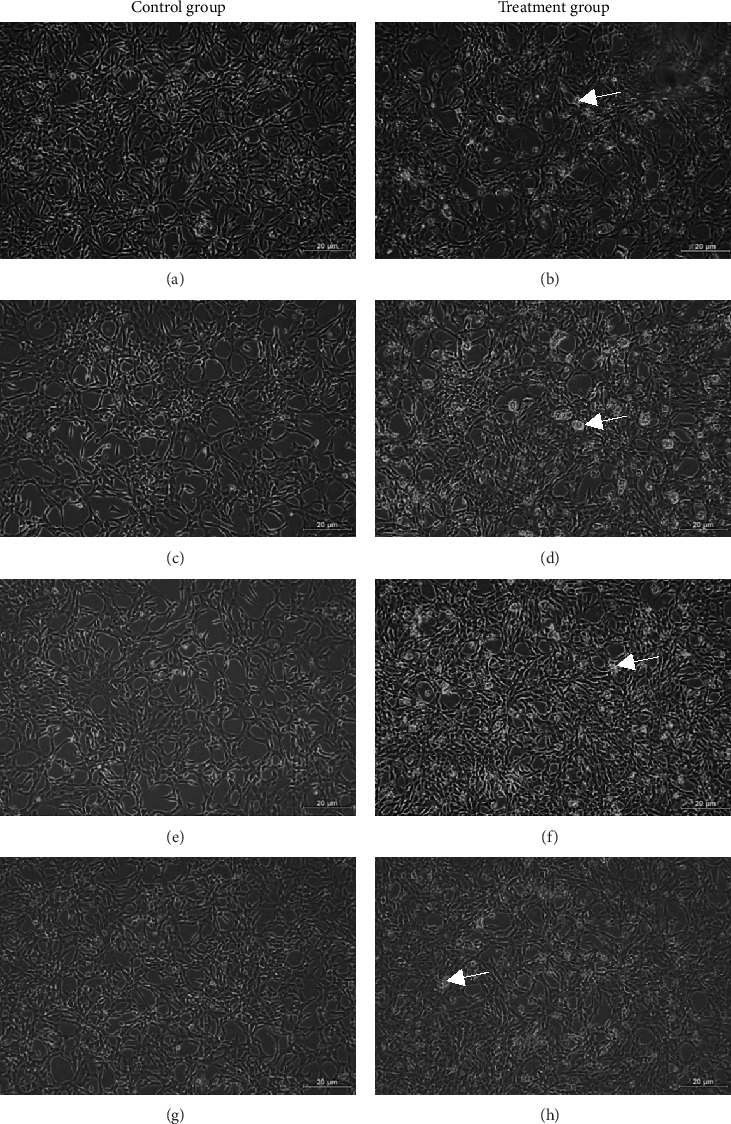
SH-SY5Y neuroblastoma cells morphology observed under phase contrast microscopy in treatment and control group after 24 h treatment. The groups are as follows: (a) vehicle control (VC), (b) Aβ only (20 μM Aβ), (c) RepSox only (100 nM), and (d) RepSox + Aβ (100 nM RepSox and 20 μM Aβ), (e) Galunisertib only (100 nM), and (f) Galunisertib + Aβ (100 nM Galunisertib and 20 μM Aβ), (g) Vactosertib only (100 nM), and (h) Vactosertib + Aβ (100 nM Vactosertib and 20 μM Aβ). The cells were pretreated with 100 nM of TGF-βR1 inhibitors for 4 h, followed by exposure of 20 μM of Aβ for 24 h. Untreated cells cultured in media containing 0.02% DMSO were included as VC. The morphological changes (dying and nondifferentiated cells with retracted neurites) observed in SH-SY5Y cells upon exposure to Aβ in the treatment groups at 24 h were as indicated by the arrows. The images were captured at 100x magnification (*n* = 3, three fields were taken for each sample).

Based on Figure 3, the trend in cell viability after 24 h of treatment was supported by the microscopic images of the cells' morphology. Notable cell morphological changes (dying and nondifferentiated cells with retracted neurites) were observed among the SH-SY5Y cells induced by Aβ in the treatment groups. The number of viable cells in the Aβ control group was lower compared with the VC, thereby indicating Aβ-induced neurotoxicity. However, with reference to control, similar cell confluency was observed when SH-SY5Y cells were treated with 100 nM of TGF-βR1 inhibitors (RepSox, Galunisertib, and Vactosertib), suggesting that these TGF-βR1 inhibitors did not exert cytotoxicity effect on SH-SY5Y cells. In the presence of Aβ, it was evident that the cell viability of SH-SY5Y cells pretreated with 100 nM of TGF-βR1 inhibitors was higher than that in the Aβ control group, indicating that TGF-βR1 inhibitors conferred neuroprotective effect against Aβ-induced neurotoxicity on SH-SY5Y cells.

### 3.2. Neuroprotective Effect of TGF-βR1 Inhibitors on SH-SY5Y Cells Against Aβ-Induced Microglia-Mediated Neuroinflammation

**Figure 4:**
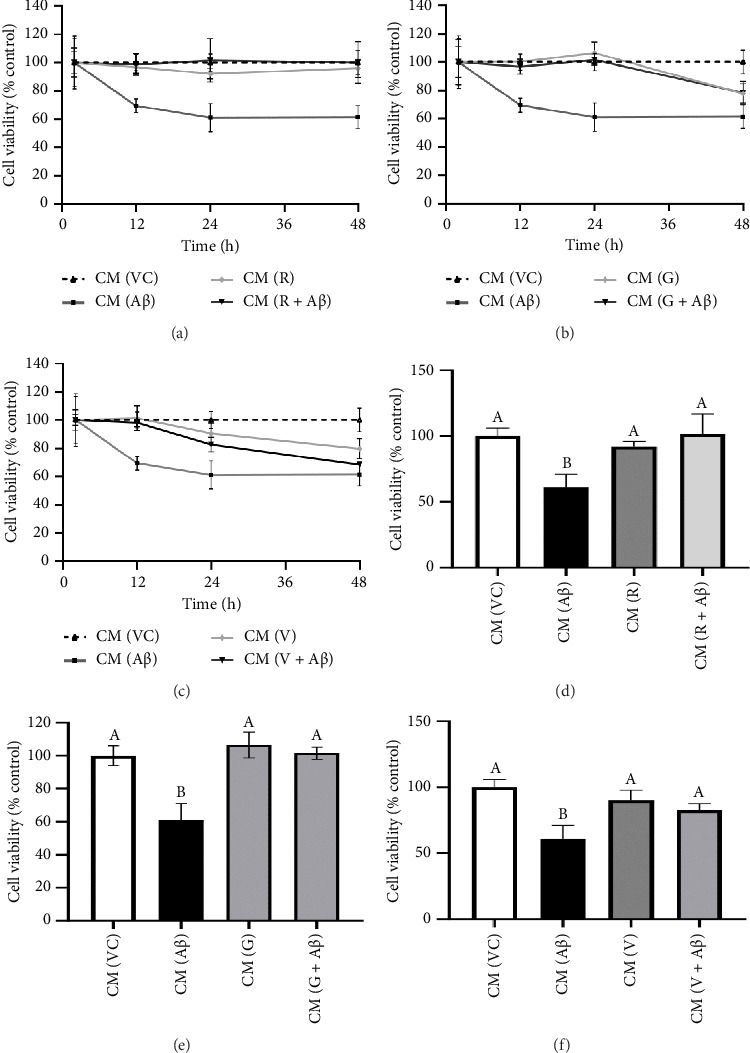
Indirect neuroprotective effect of TGF-βR1 inhibitors on SH-SY5Y neuroblastoma cells against Aβ-induced microglial-mediated neuroinflammation measured in real time over 48 h (a–c) and at the time point of *t* = 24 h (d–f). The BV-2 microglial cells were pretreated with 100 nM of TGF-βR1 inhibitors (RepSox (R) (a, d), Galunisertib (G) (b, e), and Vactosertib (V) (c, f)), respectively, for 4 h followed by the exposure of 2 μM Aβ for 24 h. Upon treatment completion, the conditioned media (CM) from the BV-2 microglia cell cultures along with the RealTime-Glo™ reagents were transferred to SH-SY5Y cells. The luminescent signal was measured at specific time points over 48 h. Media containing 0.1% DMSO was included as VC. The results were expressed as percentage cell viability versus VC. All values were shown as the mean ± SD and representative of three independent experiments (*n* = 3). The treatment groups labeled with the same alphabets are not statistically significant (*p* > 0.05).

As shown in Figures 4(a), 4(b), and 4(c), SH-SY5Y cells treated with CM from Aβ-induced BV-2 cells (CM (Aβ)) have exhibited a gradual decrease in cell viability over 48 h in comparison with cells treated with CM (VC). A significant decline in SH-SY5Y cell viability was observed at the 24-h time point in CM (Aβ) (Figures 4(d), 4(e), and 4(f); *p* < 0.05). However, SH-SY5Y cell viability in CM treated with TGF-βR1 inhibitors (CM (R/G/V) and CM (R/G/V + Aβ)) did not differ significantly in comparison with CM (VC) (Figures 4(d), 4(e), and 4(f); *p* > 0.05). Notably, the neurotoxicity effect on SH-SY5Y cells from CM of Aβ-induced BV-2 cells was significantly attenuated by pretreated TGF-βR1 inhibitors on the BV-2 cells (Figures 4(d), 4(e), and 4(f); *p* < 0.05). These findings suggest that TGF-βR1 inhibitors provide an indirect neuroprotective effect on SH-SY5Y cells against Aβ-induced microglial-mediated neuroinflammation.

### 3.3. Inhibitory Effects of TGF-βR1 Inhibitors on Aβ-Induced Proinflammatory Cytokines (TNF-α and IL-1β) Production in BV-2 Cells

**Figure 5:**
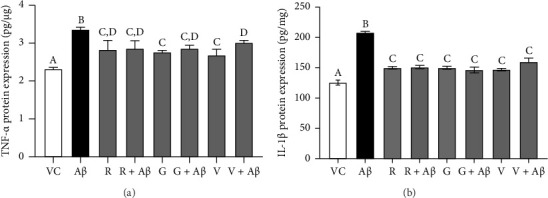
Inhibitory effects of TGF-βR1 inhibitors on TNF-α (a) and IL-1β (b) production in BV-2 microglial cells. The BV-2 microglial cells were pretreated with 100 nM of TGF-βR1 inhibitors (RepSox (R), Galunisertib (G), and Vactosertib (V)), respectively, for 4 h followed by the exposure of 2 μM Aβ for 24 h. Cytokine levels of the supernatant of BV-2 cells were measured using the ELISA kits. Media containing 0.1% DMSO were used as vehicle control (VC). Results were expressed as picograms of TNF-α per microgram of total protein (pg/μg) and picograms of IL-1β per milligram of total protein (pg/mg), respectively, and values were presented as the mean ± SEM of three independent experiments (*n* = 3). The treatment groups labeled with the same alphabets are not statistically significant (*p* > 0.05).

Based on Figure 5, Aβ-induced BV-2 cells (Aβ control group) demonstrated a significant increase in the production of TNF-α and IL-1β as compared with VC (*p* < 0.05). Conversely, pretreatment with 100 nM of TGF-βR1 inhibitors for 4 h prior to exposure of 2 μM Aβ (CM (R/G/V + Aβ)) resulted in a significant attenuation in the production of these proinflammatory cytokines compared with the Aβ control group (*p* < 0.05). These findings suggest that TGF-βR1 inhibitors confer an indirect neuroprotective effect against Aβ-induced microglial-mediated neuroinflammation by inhibiting the production of proinflammatory cytokines.

## 4. Discussion

The soluble Aβ oligomers, known as the most neurotoxic form of Aβ, are primarily responsible for Aβ-induced neurotoxicity in AD [50–52]. In the present study, the exposure of SH-SY5Y cells to 20 μM Aβsignificantly reduced the cell viability over time in comparison with the VC (Figure 2), implying the neurotoxicity effect of Aβ on neuronal cells. Interestingly, pretreatment with selective TGF-βR1 inhibitors (RepSox, Galunisertib, and Vactosertib) prior to Aβ induction have significantly improved the viability of Aβ-induced SH-SY5Y cells. This indicated that the inhibition of TGF-β signaling pathway via TGF-βR1 inhibitors confer direct neuroprotective effect against Aβ-induced neurotoxicity although the exact underlying mechanism is yet to be investigated. This aligns with the recent findings whereby, silencing of the *TGFBR1* gene has resulted in neuronal survival from Aβ-induced neurotoxicity [20]. Importantly, Aβ_1-42_ exposure reportedly increased TGF-β1 expression, resulting in neuronal apoptosis and hence promoting Aβ-mediated neurodegeneration [53]. Increased TGF-β1 levels were also observed in AD patients [54], where aberrant TGF-β signaling was found to induce self-aggregation of TGF-β1-induced antiapoptotic factor (TIAF1), causing formation of Aβ plaques in AD [55]. Taken together, these findings support the neurotoxic role of TGF-β in AD pathogenesis.

On the other hand, in the indirect neuroprotective study, SH-SY5Y cells exposed to CM from Aβ-induced BV-2 microglial cells showed a significant reduction in the cell viability compared to CM (VC) (Figure 4). This reduction of cell viability was accompanied by a significant increase in the production of both TNF-α and IL-1β in Aβ-induced BV-2 microglia cells (Figure 5). These observations were consistent with previous studies which demonstrated that overactivation of microglia due to Aβ aggregates exposure, resulted in the release of inflammatory mediators, such as TNF-α and IL-1β, which ultimately cause neuronal death [56]. In addition, these proinflammatory mediators produced by microglial cells can trigger the astrocytes to create a more inflammatory environment that could further activate the microglia, resulting in a self-perpetuating viscous cycle of neuroinflammation resulting in neuronal cell death via apoptosis [12, 57, 58]. In fact, the binding of proinflammatory cytokines to their respective receptors stimulates a signaling pathway leading to neurotoxic outcomes has been implicated in AD [13, 59]. Furthermore, the intracellular Aβ could directly activate the p-53 promoter, leading to p53-dependent apoptosis [60], which might represent the underlying mechanism of Aβ-induced microglia-mediated neurotoxicity that warrants further research studies. Moreover, in the present study, the viability of SH-SY5Y cells in CM (R/G/V + Aβ) treatment groups were markedly increased in comparison with the CM (Aβ) control group (Figure 4). These findings indicated that the inhibition of TGF-β signaling pathway in microglia cells conferred indirect neuroprotective effects on SH-SY5Y cells against Aβ-induced microglia-mediated neuroinflammation. Microglia plays an important role in regulating neuroinflammation via the production of TNF-α and IL-1β. A study by [61], had shown that Aβ-stimulated microglia secretes TNFα to induce neuronal death and this process involves iNOS activity and peroxynitrite production. In addition, it is reported that in the presence of IL-1β, microglia are activated, proliferated, and contributed to Aβ removal, enhancing the number of microglia that are available for Aβ clearance [62]. In this study, it was also found that the pretreatment with TGF-βR1 inhibitors significantly reduced the production of proinflammatory cytokines (IL-1β and TNF-α) in Aβ-induced BV-2 microglia cells (in R/G/V + Aβ treatment groups) when compared with the Aβ control group (Figure 5). This might be due to the decrease in microglia proliferation through the inhibition of TGF-β signaling, which might have resulted in the reduction of neuroinflammation and a decrease in the production of TNF-α and IL-1β [34]. Indeed, TGF-β was found to be an important factor in the regulation of microglia-mediated neuroinflammatory responses [63, 64]. A recent study also showed that by antagonizing the TGF-β signaling, the risk of microgliosis can be reduced, which may alleviate the neuronal injury due to the reduced microglial release of cytokines [34]. In addition, recent research supported the present study by demonstrating that deleting microglial apolipoprotein (APOE4) restored the induction of neurodegenerative microglial (MGnD) response in mice with amyloid and tau pathology. This led to reduced plaque formation, increased association of microglia and astrocytes with Aβ plaques, and improved neuronal survival. Moreover, APOE4 contributes to AD pathology by activating TGF-β-dependent microglial homeostatic regulators, which disrupt the crosstalk between microglia and astrocytes during neurodegeneration. Taken together, the study suggests that targeting the microglial APOE4-ITGB8-TGFβ pathway could potentially restore the MGnD microglial phenotype, which provides a promising therapeutic target in AD [65]. In preclinical models, the TGF- β targeting agents have shown promising results. The TβR1 kinase inhibitor galunisertub has been tested as a potential treatment for unresectable pancreatic cancer and advanced hepatocellular carcinoma in Phase II clinical trial. The treated group showed improved overall survival. However, TGF-β could suppress tumor in normal tissues and early stage of cancer while promote tumor growth in late stage. This biphasic role of TGF- β could result in unwanted side effects but the side effects can be mitigated by intermittent dosing and patient selection [29]. Given the notable therapeutic effects of TβR kinase inhibitors in cancer patients, targeting TGF-β signaling could be an alternative approach to selectively modulate Aβ-induced neuroinflammatory responses in microglia of AD patients.

It is important to note that the etiology of AD is multifactorial, and there are several other mechanisms and/or intracellular signaling pathways that are relevant to the pathogenesis of AD [59]. Notably, the neurons have shown to involve in inflammasome-mediated neuroinflammation independently while the nod-like receptor protein 1 (NLRP1) is found to be highly expressed in oligodendrocytes and pyramidal neuron [49, 64]. A recent mutagenesis screen revealed that silencing the TGFBR1 gene allowed neuronal survival from Aβ-induced NLRP1 inflammasome-mediated neurotoxicity [20]. Furthermore, inflammasome-mediated pyroptosis involving NLRP1 is shown to be one of the underlying mechanisms involved in the hyperphosphorylated tau protein-induced neurotoxicity [67]. Overexpression of interleukin (IL)-1β, IL-18, and the cytokines produced downstream of the inflammasome pathway and had contributed to tau hyperphosphorylation by activating tau kinases (p38 mitogen-activated protein kinase (MAPK) and glycogen synthase kinase-3beta (GSK-3β)) [68] as well as by upregulating GSK-3β and cyclin-dependent kinase 5 (CDK5) expression [69], respectively. Therefore, it is suggested that Aβ-induced NLRP1 inflammasome activation might occur upon TGF-β stimulation on the neuronal cells [20]. In addition, since TGF-β1 could also activate p38-MAPK via the Smad-independent pathway [24, 25, 70–72], RepSox and Vactosertib could inhibit p38-MAPK activity via the inhibition of TGF-βR1 receptor and TGF-β signaling [42]. Hence, future studies should focus on investigating the underlying neuroprotective mechanism of the TGF-βR1 inhibitors in AD pathogenesis through the blockade of the TGF-β signaling pathway in relation to the NLRP1 inflammasome pathway and Aβ-induced tau hyperphosphorylation.

One of the limitations of this study is the limited knowledge availability toward the mechanism of TGF-βR1 inhibitor on the direct and indirect neuroprotective effect against SH-SY5Y cells. In this study, we have only focused on one mechanism; neurotoxicity mediated by pro-inflammatory cytokines released by microglia, using SH-SY5Y cells and BV-2 cells as in vitro model. However, this model is unable to precisely replicate the pathophysiology of AD, the environment of AD brain, or the feature of central nervous system (CNS) in whole. An in vivo model that closely resembles the pathophysiology of AD should be utilized for future studies to confirm the underlying molecular mechanism of TGF-βR1 inhibitors in AD. However, translating in vitro findings to in vivo models could present several challenges, including the complexity of biological environments, where tissue architecture and interactions can affect inhibitor efficacy. Pharmacokinetics and pharmacodynamics studies are further needed in determining drug concentration and action duration. Potential side effects, such as off-target effects and immune responses, may emerge in vivo. Appropriate dosing and administration methods must be established, as effective in vitro doses may not translate directly.

## 5. Conclusion

In conclusion, TGF-βR1 inhibitors (RepSox, Galunisertib, and Vactosertib) exhibited neuroprotective effect against Aβ-induced direct neurotoxicity and microglia-mediated neuroinflammation through the suppression of proinflammatory cytokines (TNF-α and IL-1β) production in microglia cells. Hence, a treatment strategy targeting the TGF-β signaling pathway in neuronal cells and microglia cells could provide a promising therapeutic approach in AD treatment. However, further research studies including in vivo studies are required to determine the role of TGF-βR1 inhibitors by investigating its underlying neuroprotective mechanism against Aβ-induced neurotoxicity via Aβ-induced neuronal NLRP1 inflammasome-mediated neurotoxicity pathway as well as the regulation of the expression of pyroptosis-related proteins (NLRP1, IL-18, IL-1β, and caspase-1) and tau kinases including p38-MAPK and GSK-3β in Aβ-induced neuronal cells.

## Figures and Tables

**Table 1:** Treatment/control groups to access the neuroprotective effect of TGF-βR1 inhibitors against Aβ-induced direct neurotoxicity.

Group	Description
Control	
VC	SH-SY5Y cells in 0.02% DMSO, vehicle control
Aβ	20 μM Aβ-induced SH-SY5Y cells
R1/G1/V1	150 nM TGF-βR1 inhibitors-treated SH-SY5Y cells
R2/G2/V2	100 nM TGF-βR1 inhibitors-treated SH-SY5Y cells
R3/G3/V3	50 nM TGF-βR1 inhibitors-treated SH-SY5Y cells
Treatment	
R1/G1/V1 + Aβ	Aβ-induced SH-SY5Y cells pretreated with 150 nM TGF-βR1 inhibitors
R2/G2/V2 + Aβ	Aβ-induced SH-SY5Y cells pretreated with 100 nM TGF-βR1 inhibitors
R3/G3/V3 + Aβ	Aβ-induced SH-SY5Y cells pretreated with 50 nM TGF-βR1 inhibitors

**Table 2:** Conditioned media used to assess the neuroprotective effect of TGF-βR1 inhibitors against Aβ-induced microglia-mediated neuroinflammation in SH-SY5Y cells.

Group	Description
Control	
CM (VC)	CM from BV-2 cells cultured in media with 0.1% DMSO, vehicle control
CM (Aβ)	CM from BV-2 cells treated with 2 μM Aβ
CM (R/G/V)	CM from BV-2 cells treated with 100 nM TGF-βR1 inhibitors
Treatment	
CM (R/G/V + Aβ)	CM from Aβ-induced BV-2 cells pretreated with 100 nM TGF-βR1 inhibitors

**Table 3:** Treatment/control groups to assess the effect of TGF-βR1 inhibitors on the production of TNF-α and IL-1β in BV-2 cells.

Group	Description
Control	
VC	BV-2 cells in 0.1% DMSO, vehicle control
Aβ	BV-2 cells treated with 2 μM Aβ
R/G/V	BV-2 cells treated with 100 nM TGF-βR1 inhibitors
Treatment	
R/G/V + Aβ	Aβ-induced BV-2 cells pretreated with 100 nM TGF-βR1 inhibitors

## Data Availability

The datasets used and/or analyzed during the present study are available from the corresponding author upon reasonable request.
